# Cardiac-Specific AI Reconstruction Versus Hybrid Iterative Reconstruction for Emergency Coronary CT Angiography: A Paired Single-Center Study Including Triple-Rule-Out CT

**DOI:** 10.3390/jcm15207940

**Published:** 2026-10-14

**Authors:** Sung Min Ko, Hyunjung Kim, Sung-Jin Cha, Yongfeng Gao

**Affiliations:** 1 Department of Radiology, Wonju Severance Christian Hospital, Yonsei University Wonju College of Medicine, 20 Ilsan-ro, Wonju 26426, Republic of Koreacktjdwls9612@naver.com (S.-J.C.); 2 United Imaging Healthcare North America, Houston, TX 77054, USA; yongfeng.gao@united-imaging.com

**Keywords:** coronary computed tomography angiography, artificial intelligence, deep-learning reconstruction, image quality, triple-rule-out computed tomography angiography

## Abstract

**Background/Objectives:** Optimizing coronary image quality is important in emergency computed tomography (CT), including coronary CT angiography (CCTA) and triple-rule-out CT angiography (TRO-CTA). We compared objective and subjective image quality between cardiac-specific artificial intelligence (AI) reconstruction and hybrid iterative reconstruction (HIR) using paired images from the same acquisition. **Methods:** This retrospective single-center study included 66 of 75 consecutively assessed emergency examinations: 28 standard CCTA and 38 TRO-CTA examinations acquired without pharmacologic heart-rate control. Both protocols used identical cardiac acquisition settings. AI and HIR images were reconstructed from the same acquisition with matched cardiac phases and motion-correction settings. Objective measurements were obtained at nine coronary locations, and two readers independently assessed overall image quality and perceived vessel-edge sharpness in unmasked paired comparisons. **Results:** AI reduced coronary noise from 45.8 ± 8.5 to 24.2 ± 7.5 Hounsfield units (HU; −47.3%), increased signal-to-noise ratio (SNR) from 9.0 ± 2.4 to 20.6 ± 5.6 (+129.6%), and increased contrast-to-noise ratio (CNR) from 14.8 ± 4.2 to 45.8 ± 16.4 (+209.7%; all Holm-adjusted *p* < 0.001). Readers 1 and 2 preferred AI for overall quality in 65/66 and 63/66 examinations and for sharpness in 64/66 and 63/66, respectively. **Conclusions:** Cardiac-specific AI reconstruction reduced image noise and increased SNR and CNR in emergency CCTA, including TRO-CTA, with readers generally favoring AI images for perceived quality and sharpness. Further studies should evaluate whether these image-quality improvements translate into better diagnostic performance.

## 1. Introduction

Coronary computed tomography (CT) angiography (CCTA) is an established noninvasive method for detecting and characterizing coronary artery disease (CAD) and is increasingly used as an anatomic gatekeeper in patients with suspected disease [1,2]. Adequate coronary enhancement, low image noise, and clear depiction of small moving structures are important components of image quality. Achieving these conditions can be challenging in emergency practice, where higher heart rates, larger body habitus, extensive calcification, and limited breath-holding capacity are common.

Hybrid iterative reconstruction (HIR) reduces noise relative to filtered back projection, although higher reconstruction strength may alter image texture and the balance between noise suppression and spatial detail [3,4]. Artificial intelligence (AI)-based deep-learning reconstruction (DLR) algorithms have been developed to learn mappings between noisy and higher-quality CT data. Studies using several vendor platforms have demonstrated lower noise, higher signal-to-noise ratio (SNR) and contrast-to-noise ratio (CNR), or improved coronary sharpness; some prospective studies have also evaluated radiation- or contrast-dose reduction [5,6,7,8,9,10,11,12,13,14,15,16,17]. Quantifying these image-quality effects under a defined acquisition protocol provides a technical basis for evaluating reconstruction performance.

Triple-rule-out CT angiography (TRO-CTA) combines evaluation of coronary, pulmonary, and aortic causes of acute chest pain [18,19]. Our institution uses a sequential helical–axial–helical TRO-CTA protocol whose electrocardiography (ECG)-gated coronary component uses the same cardiac acquisition settings as standard CCTA [19]. Both examinations are performed using predefined acquisition and contrast-injection protocols without pharmacologic heart-rate control. This emergency workflow provides an opportunity to evaluate cardiac-specific AI reconstruction across a range of patient and imaging conditions while comparing reconstructions from identical source data within each patient.

We therefore performed a paired study comparing cardiac-specific AI reconstruction with HIR in emergency CCTA, including the coronary component of TRO-CTA. The primary objective was to quantify patient-level differences in coronary noise, SNR, and CNR. Secondary analyses assessed attenuation, independent reader preferences for overall image quality and perceived vessel-edge sharpness, region-of-interest (ROI)-specific effects, and exploratory variation across protocols and patient subgroups. The study focused on reconstruction-related image quality; it was not designed as a comparison of the clinical performance of CCTA and TRO-CTA.

## 2. Materials and Methods

### 2.1. Study Design and Population

**Figure 1:**
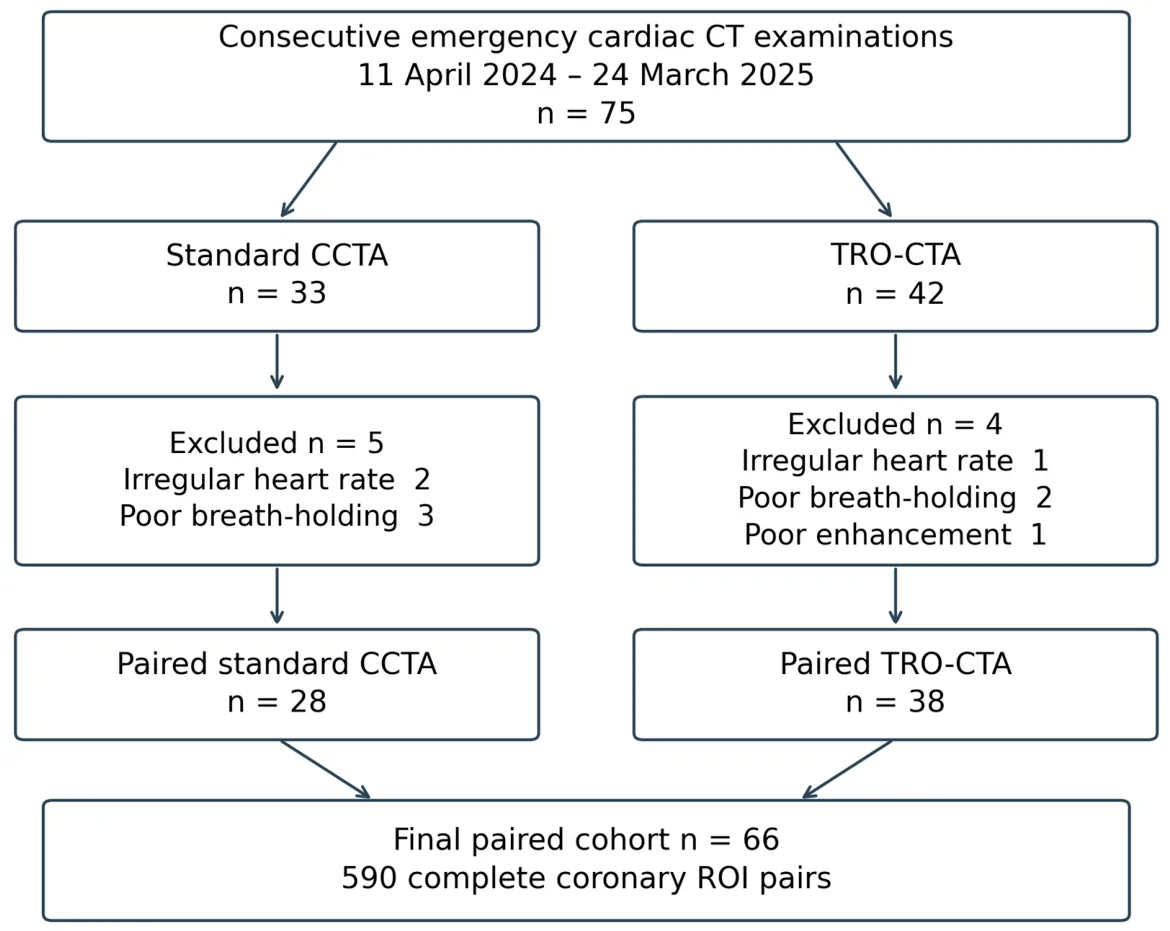
Patient selection for the paired reconstruction analysis. Counts show consecutive examinations considered, protocol-specific image-quality exclusions, and the final cohort with both reconstructions. Within the 66 retained examinations, two coronary ROIs were unmeasurable because of calcification and two distal locations were anatomically absent. These ROI omissions are separate from examination-level exclusions. CT, computed tomography; CCTA, coronary computed tomography angiography; ROI, region of interest; TRO-CTA, triple-rule-out computed tomography angiography.

This retrospective single-center paired image-quality study was conducted at Wonju Severance Christian Hospital and followed the Strengthening the Reporting of Observational Studies in Epidemiology (STROBE) reporting principles [20]. Consecutive emergency-department cardiac CT examinations performed between 11 April 2024 and 24 March 2025 were considered. Of 75 examinations (33 standard CCTA and 42 TRO-CTA), nine were excluded because irregular heart rate, inadequate breath-holding, or poor contrast enhancement prevented inclusion in the paired image-quality analysis. Standard CCTA exclusions comprised two examinations with markedly irregular heart rate and three with poor breath-holding. TRO-CTA exclusions comprised one with markedly irregular heart rate, two with poor breath-holding, and one with poor enhancement. The final cohort included 66 patients with paired AI and HIR reconstructions and matching clinical data: 28 standard CCTA and 38 TRO-CTA (Figure 1). For TRO-CTA, only the prospectively ECG-triggered coronary acquisition was analyzed.

Patients were evaluated in the emergency department for acute chest pain with or without dyspnea. Routine contraindications to iodinated contrast-enhanced CT included severe renal dysfunction, previous severe contrast reaction, uncontrolled hyperthyroidism, inability to follow breathing instructions, and clinical instability. Demographic variables, risk factors, previous coronary revascularization, coronary calcium score, and radiation-dose indices were abstracted from the clinical database. Patients were classified by a cardiac radiologist as having significant CAD (maximum coronary diameter stenosis ≥50%), nonsignificant CAD (coronary plaque with maximum diameter stenosis <50%), or no CAD (absence of coronary plaque). The study was approved by the Institutional Review Board of Wonju Severance Christian Hospital (CR324049), which waived the requirement for informed consent because of the retrospective design.

### 2.2. CT Acquisition and Reconstruction

All examinations were performed on a wide-area detector CT system (uCT 960+; United Imaging Healthcare, Shanghai, China; software uExceed R001). Both protocols used prospective ECG-triggered axial coronary acquisition with 320 detector rows of 0.5 mm, 100 kVp, a fixed tube current–time product of 180 mAs, 0.25 s gantry rotation, and a 20–80% R–R acquisition window. The least-motion phase was selected and held identical for both reconstructions: mid-diastole in 47 patients and end-systole in 19. CardioCapture motion correction at setting 5 was applied consistently to the paired reconstructions. No beta-blocker or other pharmacologic heart-rate-control agent was administered in this emergency workflow. All 66 included patients received sublingual nitroglycerin before coronary acquisition.

Both protocols used iohexol 350 mg iodine/mL (Hexosure 350; Pharvis Pharmaceutical, Seoul, Republic of Korea). Standard CCTA used 80 mL of contrast material at 5 mL/s, followed by 30 mL of saline at the same rate. Automated bolus tracking used a monitoring ROI in the descending thoracic aorta; acquisition began 6.8 s after attenuation reached 120 Hounsfield units (HU).

TRO-CTA used the sequential pulmonary–coronary–aortic protocol [19] with individualized test-bolus timing. A 15 mL contrast test injection followed by 25 mL of saline was delivered at 5 mL/s, with time to peak measured in the pulmonary trunk and descending thoracic aorta at the carina. The diagnostic injection comprised 60 mL of contrast, 30 mL of a 60:40 contrast–saline mixture (18 mL contrast and 12 mL saline), and 40 mL of saline, each at 5 mL/s. Thus, the protocol contrast volume was 78 mL for the diagnostic injection and 93 mL including the test bolus. The coronary acquisition began approximately 10 s after the completion of the pulmonary acquisition. Reported volume CT dose index (CTDIvol) and dose-length product (DLP) refer only to the analyzed coronary acquisition, not the complete TRO-CTA examination. Contrast volumes describe the specified injection protocols rather than a statistical summary of individual injection records.

Each coronary acquisition was reconstructed with CardioBoost (AI/DLR; United Imaging Healthcare) at noise-reduction level 2 and KARL3D (HIR) at strength 6 with the C_SOFT_DA kernel [17,21]. A separate reconstruction kernel was not selectable for CardioBoost. These manufacturer-recommended configurations were used in the clinical workflow and retained uniformly for this comparison; the study did not optimize strengths or compare all available configurations. Both series used a 512 × 512 matrix, 0.5 mm slice thickness, and 0.25 mm increment. Identical cardiac phases and motion-correction settings were used so that these factors did not differ within a pair. The reconstruction field of view (FOV) was typically 140 × 140 mm and was adjusted according to the patient’s heart size. Image data were transferred to an offline workstation (Vitrea, version 7.12; Vital Images, Minnetonka, MN, USA) for postprocessing. Image review and ROI measurements were performed using a picture archiving and communication system (PACS; ZeTTA PACS, version 2.0.3.10; TaeYoung Soft, Gwacheon, Republic of Korea). Acquisition and reconstruction parameters are summarized in Table S6.

### 2.3. Objective Image-Quality Analysis

Nine coronary locations were assessed: left main; proximal, mid, and distal right coronary artery (RCA); proximal, mid, and distal left anterior descending artery (LAD); and proximal and distal left circumflex artery (LCx). Two cardiac radiologists with 24 and 7 years of experience jointly selected ROIs by consensus. Coronary measurements used multiplanar reformations; the reference pericardial-fat measurement used a transaxial image. Paired series were displayed side by side, and PACS copy-and-paste tools maintained identical ROI position and area on corresponding planes. Coronary ROIs were made as large as possible within the lumen while excluding vessel wall, calcification, stents, motion artifact, and partial-volume contamination. Their size therefore varied with vessel caliber; the recorded ROI area was 7.0 ± 3.1 mm^2^. The paired pericardial-fat ROI was also placed at the same anatomical location and with the same area. Each ROI was measured once per reconstruction; independent reader measurements and repeated placements were not obtained.

Objective measurements were performed with the reconstruction method visible. Matched ROI placement standardized anatomical sampling within each image pair. The measurements characterized opacified coronary lumen while avoiding structures that could contaminate attenuation and noise estimates. If either reconstruction lacked a valid attenuation or noise measurement at a location, that entire pair was omitted from all four metrics. Anatomically absent distal segments were distinguished from technically unmeasurable locations. Patient-level means used the same available paired locations for both reconstructions.

Attenuation was the mean CT number in HU, and noise was the within-ROI standard deviation (SD). At each coronary location, SNR = coronary attenuation/coronary SD and CNR = (coronary attenuation − pericardial-fat attenuation)/pericardial-fat SD. Patient-level values were arithmetic means across complete paired coronary locations. Pericardial-fat noise and the coronary-to-fat attenuation difference were evaluated separately to identify the contributions of numerator and denominator changes. Exploratory sensitivity indices retained the coronary-to-fat attenuation difference but replaced the fat-noise denominator with aortic or left-ventricular blood-pool SD to assess dependence on the noise reference.

### 2.4. Independent Subjective Image Quality and Sharpness Assessments

The original evaluation retained a consensus overall preference score. During manuscript revision, the same two radiologists independently reassessed the paired examinations and retained separate scores for overall image quality and perceived vessel-edge sharpness, defined as clarity of the coronary lumen boundary. The comparative scale was +1 for AI superior, 0 for no preference, and −1 for HIR superior. Each criterion was scored once per examination rather than by segment. These independent ratings replaced the original consensus score in the principal subjective analysis, without consensus adjudication. Images were displayed side by side with algorithm identity visible; presentation was not randomized, and a controlled washout interval was not documented. The assessment measured comparative visual preference, including perceived sharpness, rather than quantitative spatial resolution or segment-level diagnostic adequacy.

### 2.5. Statistical Analysis

The patient was the primary unit of analysis. Continuous variables are reported as mean ± SD, except calcium score, reported as median and interquartile range (IQR); categorical variables are counts and percentages. Patient-level AI–HIR differences were evaluated using paired *t*-tests, paired mean differences with nominal 95% confidence intervals (CIs), and Cohen *d*_z_. Holm adjustment controlled multiplicity across the three co-primary outcomes (noise, SNR, CNR). Attenuation and denominator-related analyses were secondary or exploratory. Wilcoxon signed-rank tests assessed sensitivity to distributional assumptions. Arithmetic percentage change was calculated as 100 × (mean AI − mean HIR)/mean HIR. No equivalence or noninferiority margin was specified.

ROI-level linear models included reconstruction algorithm, protocol, eight location indicators, and an algorithm-by-protocol interaction. Repeated observations were handled with patient-clustered robust covariance using the finite-sample correction CR1: *G*/(*G* − 1) × (*N* − 1)/(*N* − *K*), where *G* = 66 patients, *N* = 1180 measurements, and *K* = 12 coefficients; *t*-based inference used 65 degrees of freedom. Separate ROI paired comparisons were Holm-adjusted across nine locations within each outcome. The four algorithm-by-protocol interaction tests formed a separate Holm family. Independent reader scores were summarized separately by criterion and reader. Exact agreement received Wilson 95% CIs. Unweighted Cohen κ was also calculated. For each criterion, a percentile bootstrap 95% CI used 20,000 patient-level draws (seed 20261006), excluding draws in which κ was undefined; 19,671 quality draws and 19,693 sharpness draws were valid. Because ratings were highly concentrated, κ and its bootstrap CI were interpreted cautiously alongside observed agreement and the cross-tabulations. The underlying cross-tabulations were retained. Exact two-sided sign tests, excluding ties, were exploratory and were not used to establish diagnostic superiority.

Exploratory subgroups used protocol, heart rate (<70 or ≥70 beats/min), body mass index (BMI; <25 or ≥25 kg/m^2^), and Agatston calcium score (<400 or ≥400). Their effect summaries were geometric mean percentage changes derived from AI/HIR ratios: 100 × {exp[mean ln(AI/HIR)] − 1}, with CIs derived on the log scale. These differ from arithmetic percentage changes. Three patient-level regression models of ln(AI/HIR), one each for noise, SNR, and CNR, included protocol, BMI per 5 kg/m^2^, heart rate per 10 beats/min, CTDIvol per 5 mGy, and ln(1 + calcium score). The addition of 1 retained the 30 patients with zero calcium. Type 3 heteroscedasticity-consistent covariance (HC3) and *t*-based inference with 60 degrees of freedom were used; Holm adjustment covered all 15 modifier tests across the three models. Model CIs are nominal, not simultaneous. Tests were two-sided. No a priori sample-size calculation was performed because this retrospective study included the available eligible examinations. Precision is described by CIs; nonsignificance is not evidence of subgroup equivalence. Analyses were implemented in Python 3.12 using NumPy 2.3.5, pandas 2.2.3, and SciPy 1.17.0. ChatGPT (GPT-6 Astra, OpenAI, San Francisco, CA, USA) assisted with statistical code preparation and explanation; the authors reviewed the code, analyses, and scientific interpretation.

## 3. Results

### 3.1. Study Population

The final cohort comprised 66 patients: 28 standard CCTA and 38 TRO-CTA examinations. Mean age was 63.9 ± 18.3 years, and 28 patients (42.4%) were women. Mean BMI was 23.7 ± 3.9 kg/m^2^ and mean heart rate was 74.9 ± 18.1 beats/min; 40 patients (60.6%) had heart rate ≥ 70 beats/min. Nineteen patients had BMI ≥ 25 kg/m^2^, and 19 had a calcium score ≥ 400. Median calcium score was 36.0 (IQR, 0–563.7). Protocol-stratified clinical characteristics, CAD categories, medication administration, contrast protocols, and coronary-acquisition radiation indices are shown in Table 1.

Significant CAD was present in 26 patients (39.4%), nonsignificant CAD in 16 (24.2%), and no CAD in 24 (36.4%). Corresponding counts were 10, 8, and 10 for standard CCTA and 16, 8, and 14 for TRO-CTA. Hypertension, dyslipidemia, diabetes, smoking history, and family history of CAD were recorded in 38, 28, 21, 17, and four patients, respectively. Previous percutaneous coronary intervention (PCI) or coronary stenting was recorded in five patients (7.6%): three in the standard CCTA group (10.7%) and two in the TRO-CTA group (5.3%). No patient had undergone coronary artery bypass grafting (CABG).

Of 594 possible paired coronary locations (66 × 9), 590 complete pairs were analyzed for all four metrics. Two mid-RCA locations were unmeasurable because severe calcification precluded lumen-only sampling. One distal RCA was anatomically absent because of coronary dominance, and one distal LAD was absent because a short LAD ended at the midventricular level. Thus, two exclusions reflected unmeasurable lumen regions and two reflected anatomy. Patient-level summaries were available for all 66 patients.

### 3.2. Primary Paired Image-Quality Results

**Figure 2:**
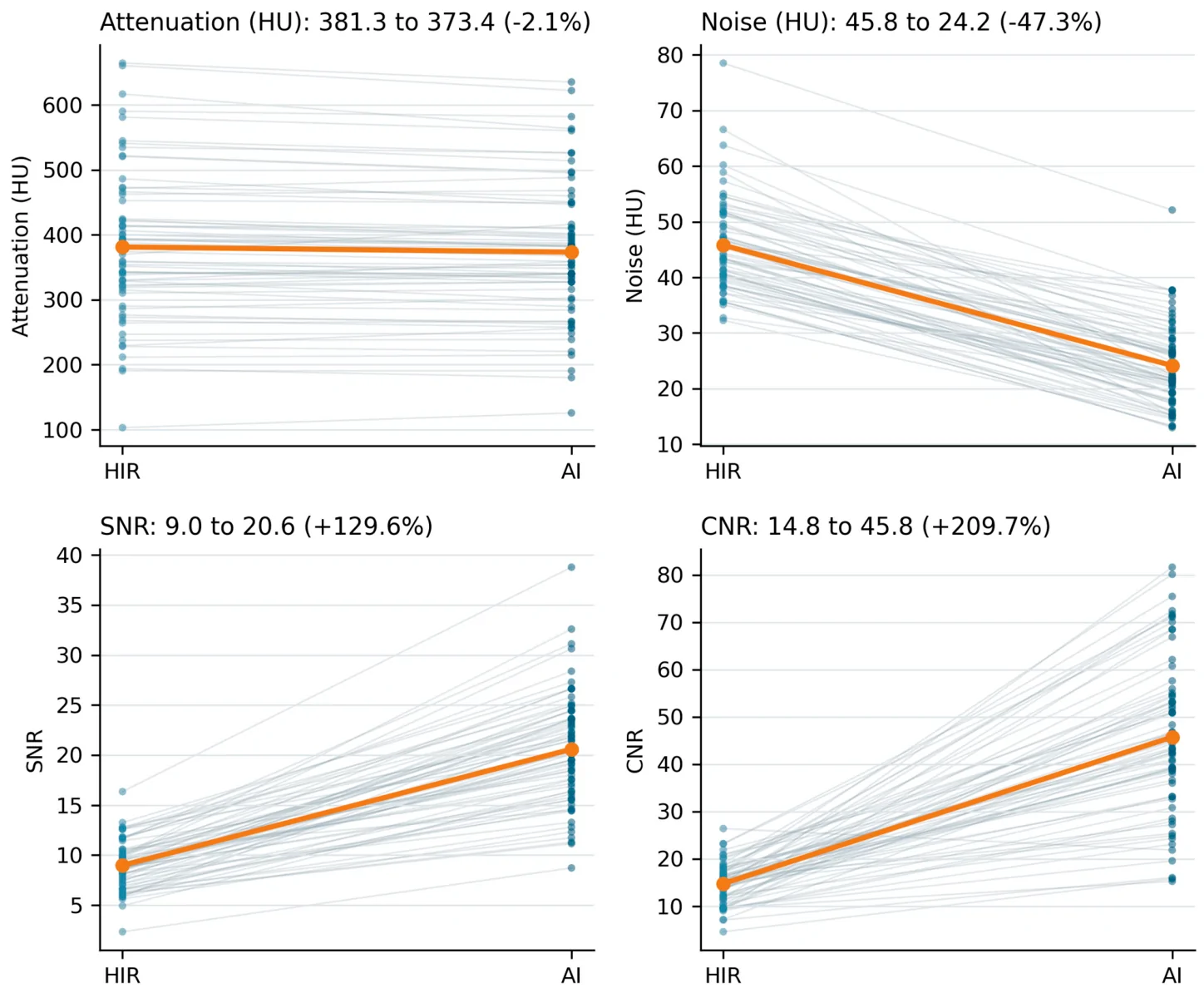
Paired patient-level coronary image-quality metrics. Each line connects HIR and AI values from the same patient; orange points and lines show arithmetic means. *n* = 66. Percentage changes are based on ratios of group arithmetic means. AI, artificial intelligence; CNR, contrast-to-noise ratio; HIR, hybrid iterative reconstruction; HU, Hounsfield units; SNR, signal-to-noise ratio.

AI reduced coronary noise from 45.8 ± 8.5 to 24.2 ± 7.5 HU (difference, −21.6 HU; 95% CI, −23.2 to −20.0; −47.3%; Cohen *d*_z_ = −3.33), increased SNR from 9.0 ± 2.4 to 20.6 ± 5.6 (difference, 11.6; 95% CI, 10.5–12.7; +129.6%; *d*_z_ = 2.66), and increased CNR from 14.8 ± 4.2 to 45.8 ± 16.4 (difference, 31.0; 95% CI, 27.2–34.8; +209.7%; *d*_z_ = 1.99). All three co-primary Holm-adjusted *p* values were <0.001; Wilcoxon tests also yielded *p* < 0.001 (Table 2; Figure 2).

Coronary attenuation decreased from 381.3 ± 113.5 to 373.4 ± 105.2 HU (difference, −7.9 HU; 95% CI, −12.1 to −3.7; −2.1%; *p* < 0.001). Pericardial-fat noise decreased by 65.8%, whereas fat attenuation showed no statistically significant difference (*p* = 0.098). The coronary-to-fat attenuation difference decreased by 5.6 HU (95% CI, −10.6 to −0.6; *p* = 0.028). The CNR increase was therefore driven primarily by reduced reference-fat noise. Alternative denominator indices increased by 164.2% using aortic noise and 182.2% using ventricular noise (Table S4), supporting the direction of the ratio-based improvement while showing that its magnitude depended on the noise reference.

### 3.3. Independent Subjective Image Quality and Sharpness

For overall quality, readers 1 and 2 preferred AI in 65/66 (98.5%) and 63/66 (95.5%) examinations, respectively. For perceived vessel-edge sharpness, the corresponding counts were 64/66 (97.0%) and 63/66 (95.5%). Neither reader preferred HIR for either criterion; all remaining ratings indicated no preference (Table 3). Exact agreement for quality was 62/66 (93.9%; 95% CI, 85.4–97.6%), with Cohen κ of −0.023 (bootstrap 95% CI, −0.055 to 0.000). Both readers favored AI in 62 examinations, but none received a no-preference rating from both readers. Expected agreement from these concentrated marginal distributions was 94.1%, slightly above observed agreement. For sharpness, exact agreement was 63/66 (95.5%; 95% CI, 87.5–98.4%), and κ was 0.377 (bootstrap 95% CI, −0.038 to 1.000). Full cross-tabulations and protocol-specific results are provided in Table S5.

### 3.4. ROI-Level and Protocol-Specific Analyses

At each of the nine coronary locations, AI significantly reduced noise and increased SNR and CNR after Holm adjustment (all adjusted *p* < 0.001; Table S1A–D). Attenuation differences were location-dependent: mean attenuation was lower with AI in the proximal RCA, mid RCA, and mid LAD after Holm adjustment, whereas the other six locations showed no adjusted difference.

Within standard CCTA, arithmetic changes were −48.6% for noise, +131.3% for SNR, and +170.6% for CNR; within TRO-CTA they were −46.3%, +128.1%, and +240.6% (Table 4). The ROI-model algorithm-by-protocol interaction estimates were 7.59 HU for attenuation (95% CI, −0.66 to 15.85; Holm *p* = 0.213), −0.19 HU for noise (−3.19 to 2.81; *p* = 0.901), −1.40 for SNR (−3.59 to 0.80; *p* = 0.416), and 8.24 for CNR (0.79–15.70; *p* = 0.123). The CNR interaction CI is nominal, explaining its exclusion of zero despite the adjusted *p* value. No interaction met the multiplicity-adjusted threshold. Full model estimates are reported in Table S2.

### 3.5. Exploratory Patient-Subgroup Analyses

**Figure 3:**
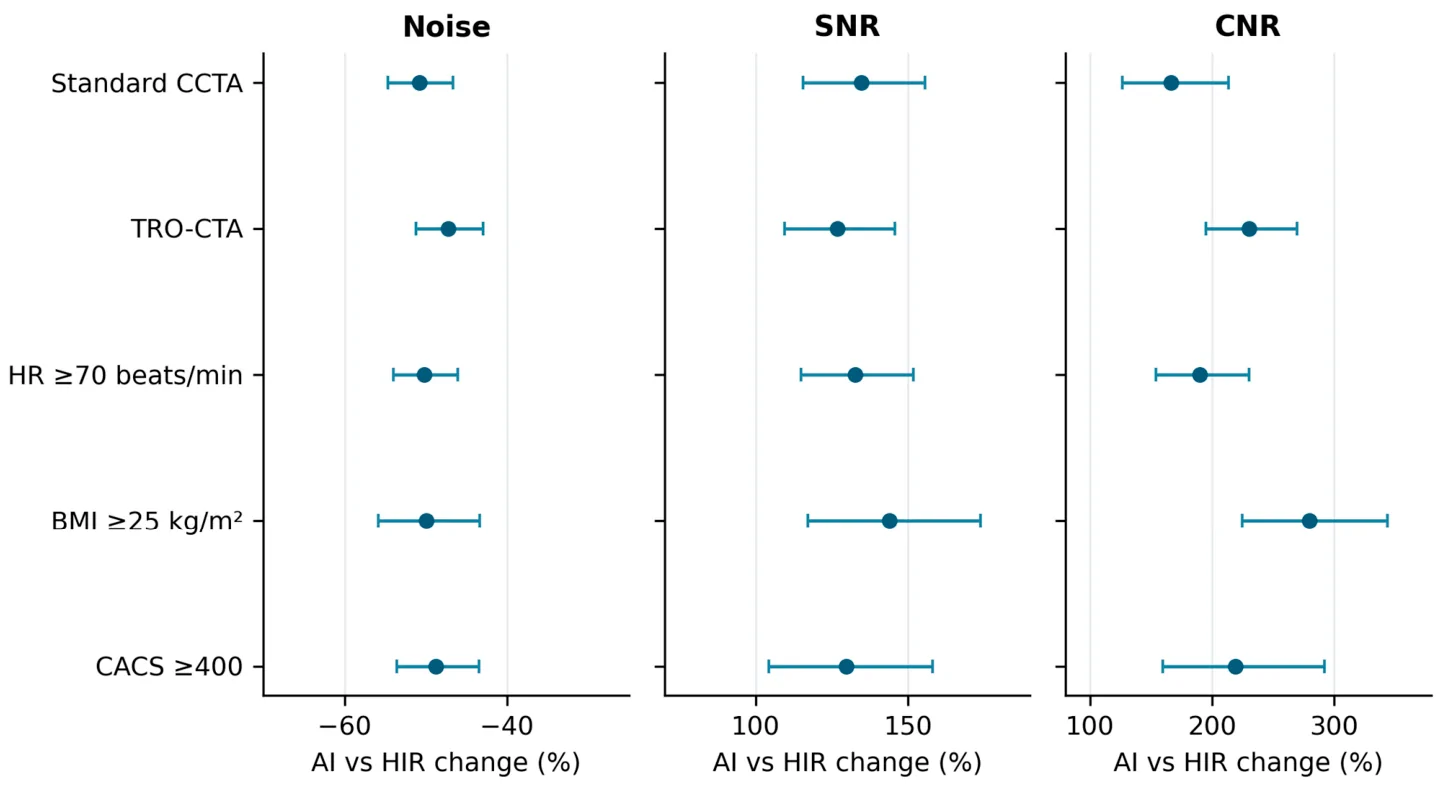
Exploratory reconstruction effects across protocols and subgroups. Points show geometric mean percentage changes for AI versus HIR; bars show nominal 95% Cis. Negative noise values indicate reduction. These are exploratory image-quality comparisons. AI, artificial intelligence; BMI, body mass index; CACS, coronary artery calcium score; CCTA, coronary computed tomography angiography; CI, confidence interval; CNR, contrast-to-noise ratio; HIR, hybrid iterative reconstruction; HR, heart rate; SNR, signal-to-noise ratio; TRO-CTA, triple-rule-out computed tomography angiography.

Noise reduction and increased SNR/CNR were observed in the higher-heart-rate, higher-BMI, and higher-calcium subgroups (Figure 3; Table 5). For calcium score ≥ 400 (*n* = 19), geometric changes were −48.7% for noise, +129.6% for SNR, and +219.1% for CNR. No modifier met the Holm-adjusted threshold across the 15 patient-level model tests (Table S3). These exploratory findings describe reconstruction-related changes in measured image-quality indices within each subgroup.

**Figure 4:**
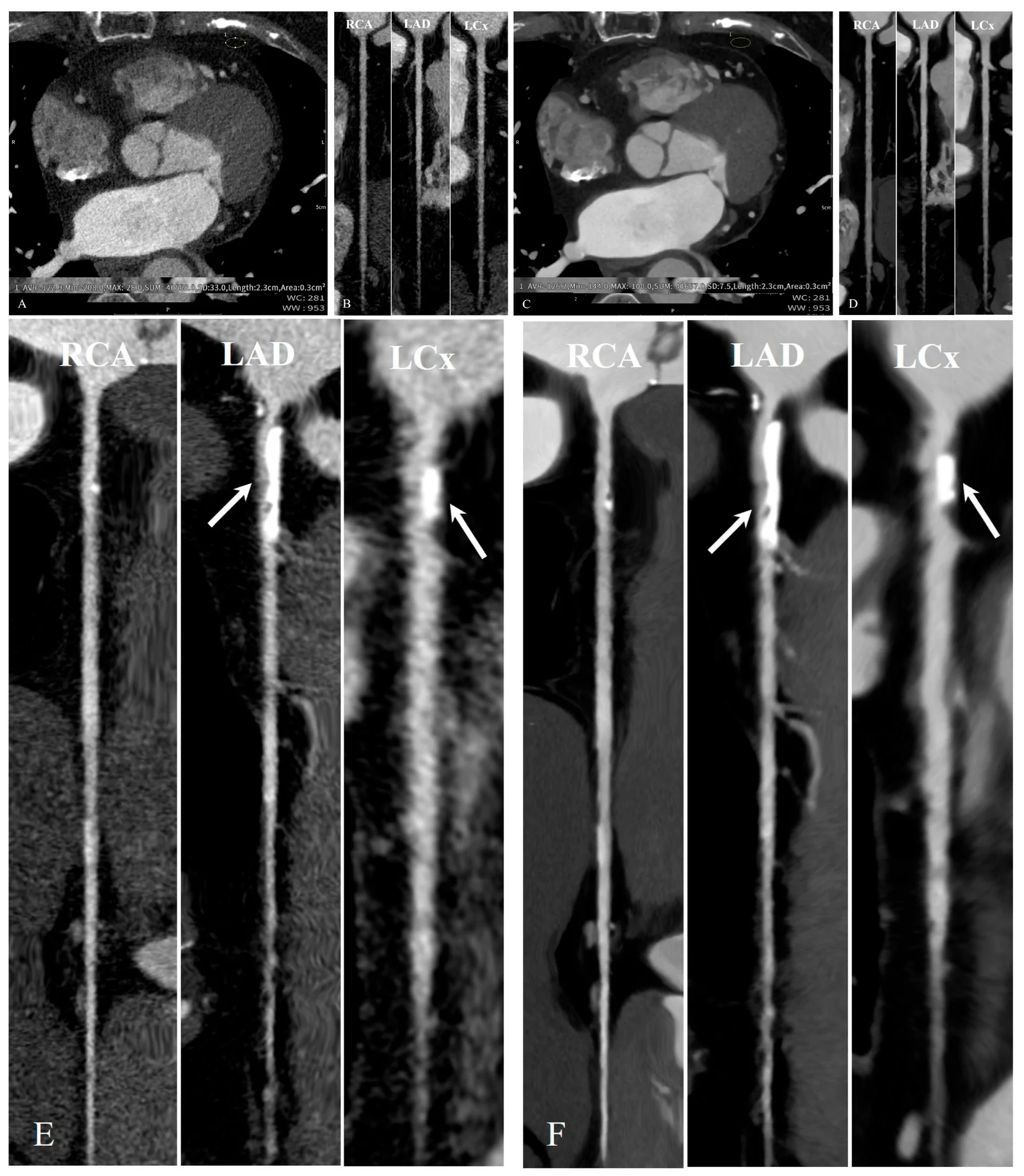

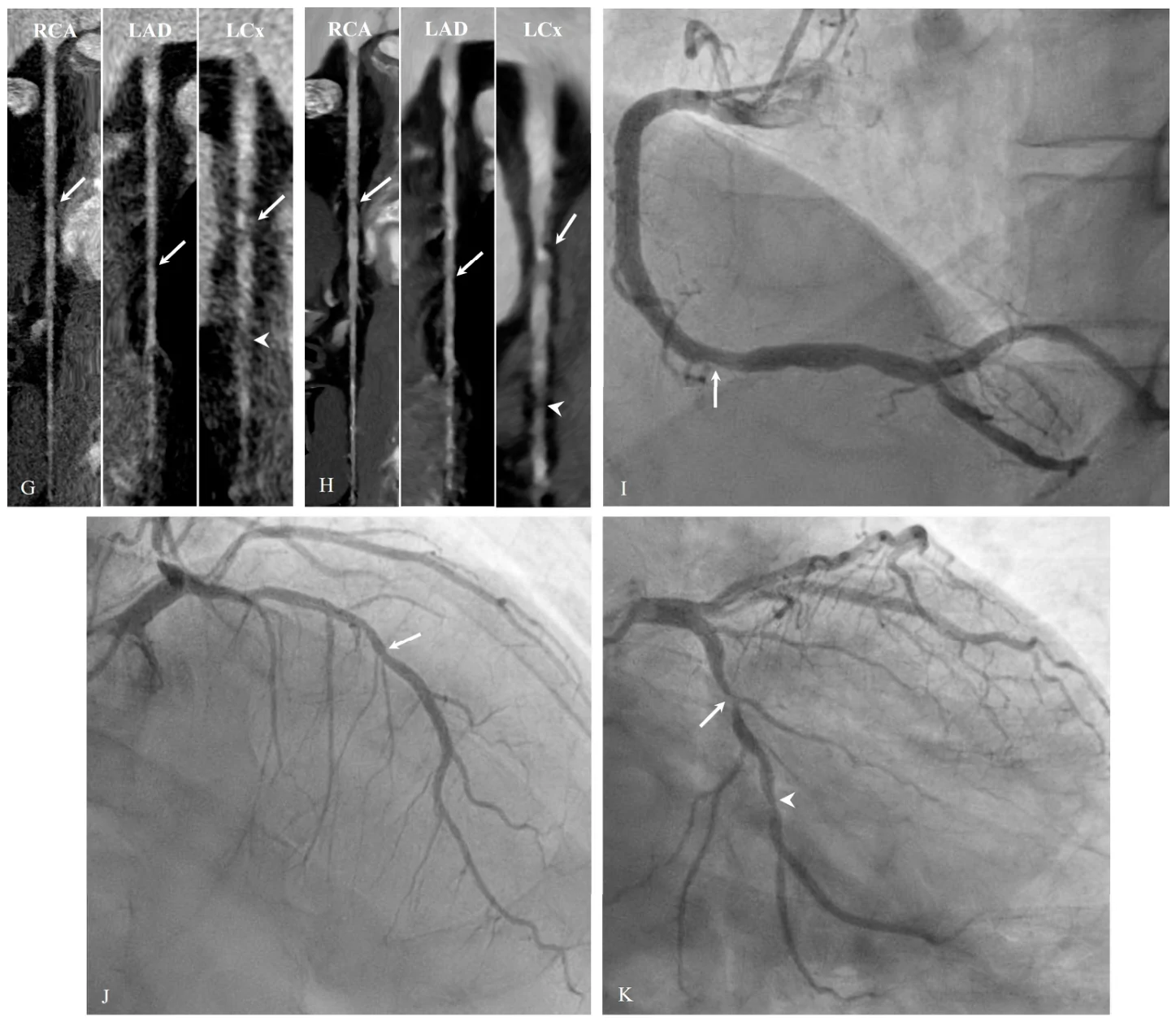
Matched ROI placement and representative coronary appearances. Case 1 (**A**–**D**) shows paired HIR (**A**,**B**) and AI (**C**,**D**) images in a patient with normal coronary arteries. Matched pericardial-fat ROIs are shown on corresponding transaxial images (**A**,**C**). Straightened multiplanar reformatted images (**B**,**D**) display the RCA, LAD, and LCx in the same order, allowing direct visual comparison of coronary image appearance between HIR and AI. Only the pericardial-fat ROI is displayed in this case. Corresponding HIR and AI images use identical plane, field of view, magnification, and window settings; the transaxial images also use the same slice position. Case 2 (**E**,**F**). HIR (**E**) and AI (**F**) images show calcified LAD and LCx segments (arrows). Paired images illustrate image noise and the appearance of the opacified lumen adjacent to calcification; calcium blooming was not quantified. Case 3 (**G**–**K**). HIR (**G**) and AI (**H**) show intermediate distal RCA and mid-LAD stenoses (arrows), and severe proximal LCx (arrow) and distal LCx (arrowhead) stenoses. Angiography illustrates the corresponding RCA (**I**), LAD (**J**), and LCx (**K**) appearances. Paired CT images use matched display geometry and window settings. The angiographic images provide anatomical context for this illustrative example; diagnostic performance was not assessed. AI, artificial intelligence; CT, computed tomography; HIR, hybrid iterative reconstruction; LAD, left anterior descending artery; LCx, left circumflex artery; RCA, right coronary artery; ROI, region of interest.

Matched pericardial-fat sampling and representative paired appearances of normal, calcified, and stenotic coronary segments are shown in Figure 4.

## 4. Discussion

In this paired emergency cardiac CT study, cardiac-specific AI reconstruction reduced coronary noise by 47.3% and increased SNR and CNR by 129.6% and 209.7%, respectively, compared with HIR. Both readers favored AI for overall image quality and perceived vessel-edge sharpness in most examinations. These findings demonstrate a substantial denoising effect under a standardized clinical workflow without pharmacologic heart-rate control. The inclusion of both standard CCTA and the coronary component of TRO-CTA extends the assessment to two emergency imaging pathways using the same cardiac acquisition settings.

A central strength is the reconstruction of both image series from the same acquisition. Within each patient, anatomy, contrast delivery, acquisition dose, cardiac phase, and motion-correction settings were held constant. Matched ROI placement further standardized anatomical sampling. Consequently, the paired differences reflect the evaluated reconstruction configurations rather than the differences between separate scans. The nine-location analysis demonstrated noise reduction throughout the assessed coronary tree, complementing the patient-level primary analysis. This design directly addresses the study objective of quantifying image-quality improvement attributable to reconstruction.

Our results complement previous DLR studies while extending their application to emergency imaging. Wu et al. prospectively compared 60-kVp CCTA using 28 mL of contrast with routine 100-kVp CCTA using 44 mL in 89 patients on the same scanner platform, evaluating CardioBoost and the consistency of coronary stenosis and CT-derived fractional flow reserve measurements [17]. Their reference was routine-dose CCTA. Our same-acquisition comparison isolates the reconstruction effect at the clinical acquisition settings and includes TRO-CTA. Takafuji et al. compared super-resolution DLR with conventional DLR in 58 patients, including quantitative vessel-sharpness assessment and an invasive-angiography comparison in 13 patients and 167 segments [13]. The present study evaluates denoising and comparative perceived sharpness; it complements those spatial-detail and diagnostic investigations without treating their endpoints as interchangeable.

The emergency setting is an important feature of this cohort. No pharmacologic heart-rate control was used, and 60.6% of patients had heart rates ≥ 70 beats/min. Both pathways used identical cardiac scan settings and predefined, protocol-specific contrast administration. Their different contrast-delivery and timing strategies can affect absolute enhancement and noise, but each AI–HIR comparison used the same source acquisition. Including both pathways therefore broadens the conditions under which the reconstruction effect was evaluated; it does not constitute a comparison of the clinical merits of CCTA and TRO-CTA. The same direction of noise, SNR, and CNR changes was observed within both protocols and the patient subgroups. The subgroup analyses remain exploratory, and nonsignificant interaction tests do not establish equivalent effect sizes.

The quantitative findings are consistent with a predominantly denoising mechanism. Coronary attenuation decreased slightly, by 7.9 HU, while the reduction in noise increased both SNR and CNR. Pericardial-fat noise decreased by 65.8%, compared with 47.3% for coronary noise, explaining the particularly large fat-referenced CNR gain. Alternative aortic and ventricular noise references also yielded higher ratio-based indices, although with different magnitudes. This sensitivity analysis supports the direction of the image-quality effect while clarifying that the percentage CNR change is specific to its reference tissue. These metrics quantify noise and contrast relative to noise; they are not direct measurements of spatial resolution.

Independent reader assessments complemented the quantitative measurements. Both readers generally favored AI for overall quality and perceived vessel-edge sharpness, with exact agreement of 93.9% and 95.5%. Because ratings were concentrated in the AI-preferred category, κ depended strongly on the marginal score distributions: overall-quality κ was slightly negative despite high observed agreement, and the sharpness estimate had a wide confidence interval. Reporting the individual scores, cross-tabulations, observed agreement, and κ together provides a fuller account of these comparative judgments. Although conspicuous differences in noise and texture can make complete masking difficult, randomized presentation with concealed algorithm labels would still reduce expectation bias and strengthen future reader studies.

Image-quality assessment is a distinct and useful objective in the evaluation of reconstruction methods. Here, the combination of matched quantitative measurements and independent visual comparisons characterizes how AI denoising changes emergency coronary images. Diagnostic assessment of stenosis, calcification, stents, and motion artifacts represents a subsequent task-specific question. Future studies can build on these findings using quantitative vessel-sharpness measurements, segment-level diagnostic-adequacy scores, and appropriate diagnostic reference standards. More broadly, AI is being investigated across cardiovascular practice, including electrocardiographic interpretation, mapping, and ablation planning, as reviewed by Cipollone et al. [22]. Reconstruction is one application within this wider field, for which image quality, diagnostic performance, and clinical impact are complementary stages of evaluation.

This study has limitations. The retrospective single-center cohort was modest, with no a priori sample-size calculation. Paired estimates and CIs describe the observed effects, but the smaller subgroups provide limited precision for effect-modification analyses. Exclusion of nine examinations for irregular heart rate, poor breath-holding, or poor enhancement limits applicability to technically compromised scans. Only one scanner and one AI/HIR setting combination were evaluated, limiting extrapolation to other systems and reconstruction configurations. Objective ROIs were placed jointly with reconstruction identity visible and measured once, precluding assessment of independent measurement reproducibility and repeatability. ROI selection could be influenced by expectation bias, and calcium, stents, and artifacts were excluded from these measurements. Subjective assessments were unmasked, without randomized presentation or a documented controlled washout interval, and are susceptible to expectation bias. Concentrated reader-score distributions also limit κ interpretation. Diagnostic accuracy, clinical outcomes, reconstruction time, and dose-reduction strategies were outside the evaluated endpoints.

## 5. Conclusions

Cardiac-specific AI reconstruction substantially reduced coronary image noise and increased SNR and CNR compared with HIR from the same acquisition in emergency CCTA, including TRO-CTA. Independent readers generally preferred AI images for overall quality and perceived vessel-edge sharpness. These findings support AI reconstruction as an image-quality enhancement approach within the evaluated emergency workflow. Further studies should evaluate whether these image-quality improvements translate into better diagnostic performance and clinical utility.

## Figures and Tables

**Table 1:** Clinical characteristics and protocol details.

Characteristic	Overall *n* = 66	Standard CCTA *n* = 28	TRO-CTA *n* = 38
Age (years)	63.9 ± 18.3	60.6 ± 20.0	66.3 ± 16.7
Female sex	28 (42.4%)	7 (25.0%)	21 (55.3%)
BMI (kg/m^2^)	23.7 ± 3.9	22.3 ± 2.8	24.7 ± 4.3
Heart rate (beats/min)	74.9 ± 18.1	73.2 ± 18.4	76.2 ± 17.9
Heart rate ≥ 70 beats/min	40 (60.6%)	16 (57.1%)	24 (63.2%)
Calcium score, median (IQR)	36.0 (0.0–563.7)	54.3 (0.0–403.8)	21.5 (0.0–619.8)
Calcium score = 0	30 (45.5%)	12 (42.9%)	18 (47.4%)
Calcium score ≥ 400	19 (28.8%)	7 (25.0%)	12 (31.6%)
Hypertension	38 (57.6%)	18 (64.3%)	20 (52.6%)
Dyslipidemia	28 (42.4%)	11 (39.3%)	17 (44.7%)
Diabetes mellitus	21 (31.8%)	9 (32.1%)	12 (31.6%)
Smoking history	17 (25.8%)	6 (21.4%)	11 (28.9%)
Family history of CAD	4 (6.1%)	0 (0.0%)	4 (10.5%)
Significant CAD	26 (39.4%)	10 (35.7%)	16 (42.1%)
Nonsignificant CAD	16 (24.2%)	8 (28.6%)	8 (21.1%)
No CAD	24 (36.4%)	10 (35.7%)	14 (36.8%)
Previous PCI/coronary stenting	5 (7.6%)	3 (10.7%)	2 (5.3%)
Previous CABG	0 (0.0%)	0 (0.0%)	0 (0.0%)
Medication, contrast protocol, and coronary radiation indices			
**Parameter**	**Overall**	**Standard CCTA**	**TRO-CTA**
Nitroglycerin	66 (100%)	28 (100%)	38 (100%)
Heart-rate-control medication	0	0	0
Protocol contrast volume (mL)	Protocol-specific	80	93 including test bolus
Diagnostic contrast volume (mL)	Protocol-specific	80	78
Tube voltage/current–time product	100 kVp/180 mAs	100 kVp/180 mAs	100 kVp/180 mAs
CTDIvol (mGy)	19.0 ± 5.3	18.2 ± 4.5	19.6 ± 5.8
DLP (mGy·cm)	303.1 ± 83.8	290.8 ± 72.0	312.2 ± 91.5

Values are mean ± SD or *n* (%) unless specified. CAD was classified by maximum coronary diameter stenosis: significant CAD, ≥50%; nonsignificant CAD, plaque with maximum stenosis < 50%; no CAD, absence of coronary plaque. Blank risk-factor fields represent absence. Protocol groups were not randomized. BMI, body mass index; CABG, coronary artery bypass grafting; CAD, coronary artery disease; CCTA, coronary computed tomography angiography; IQR, interquartile range; PCI, percutaneous coronary intervention; SD, standard deviation; TRO-CTA, triple-rule-out computed tomography angiography. Contrast values are protocol specifications. TRO-CTA contrast includes a 15 mL test bolus plus 60 mL neat contrast and 18 mL within a 30 mL mixture. Radiation indices describe the coronary acquisition only. CTDIvol, volume computed tomography dose index; DLP, dose-length product.

**Table 2:** Patient-level paired image-quality results in 66 patients.

Metric	HIR	AI	AI − HIR (95% CI)	Arithmetic Change	*p* Value
Attenuation (HU)	381.3 ± 113.5	373.4 ± 105.2	−7.9 (−12.1 to −3.7)	−2.1%	<0.001
Noise (HU)	45.8 ± 8.5	24.2 ± 7.5	−21.6 (−23.2 to −20.0)	−47.3%	<0.001
SNR	9.0 ± 2.4	20.6 ± 5.6	11.6 (10.5 to 12.7)	129.6%	<0.001
CNR	14.8 ± 4.2	45.8 ± 16.4	31.0 (27.2 to 34.8)	209.7%	<0.001
Fat attenuation (HU)	−92.0 ± 27.9	−94.4 ± 25.7	−2.3 (−5.1 to 0.4)	2.5%	0.098
Fat noise (HU)	32.9 ± 6.1	11.3 ± 4.2	−21.7 (−23.5 to −19.9)	−65.8%	<0.001
Coronary–fat difference (HU)	473.3 ± 118.6	467.7 ± 110.2	−5.6 (−10.6 to −0.6)	−1.2%	0.028

Values are mean ± SD. Patient means use identical complete paired coronary ROI sets. CIs are nominal paired-*t* intervals. *p* values are Holm-adjusted across noise, SNR, and CNR; other rows show unadjusted exploratory *p* values. Arithmetic change = 100 × (mean AI − mean HIR)/mean HIR. The positive percentage for negative fat attenuation reflects the signed denominator and should not be interpreted as increased enhancement. SD, standard deviation; AI, artificial intelligence; CI, confidence interval; CNR, contrast-to-noise ratio; HIR, hybrid iterative reconstruction; HU, Hounsfield units; ROI, region of interest; SNR, signal-to-noise ratio.

**Table 3:** Independent reader-specific comparative assessments.

Criterion	Reader	AI Superior	No Preference	HIR Superior
Overall quality	1	65 (98.5%)	1 (1.5%)	0 (0.0%)
Overall quality	2	63 (95.5%)	3 (4.5%)	0 (0.0%)
Perceived sharpness	1	64 (97.0%)	2 (3.0%)	0 (0.0%)
Perceived sharpness	2	63 (95.5%)	3 (4.5%)	0 (0.0%)
**Criterion**	**Exact agreement** (**95% CI**)		**Cohen κ** (**95% CI**)	
Overall quality	93.9% (85.4 to 97.6%)		−0.023 (−0.055 to 0.000)	
Perceived sharpness	95.5% (87.5 to 98.4%)		0.377 (−0.038 to 1.000)	

Independent unmasked reassessment during revision; *n* = 66 per reader and criterion. Scores: +1 AI superior, 0 no preference, −1 HIR superior. No preference is not a formal equivalence judgment. Agreement Cis use Wilson intervals. Κ Cis use 19,671 defined quality estimates and 19,693 defined sharpness estimates from 20,000 patient-level bootstrap draws per criterion. No consensus adjudication was applied. AI, artificial intelligence; CI, confidence interval; HIR, hybrid iterative reconstruction.

**Table 4:** Protocol-specific paired effects.

Metric	Protocol	HIR	AI	AI – HIR (95% CI)	Arithmetic Change
Attenuation (HU)	CCTA	390.6 ± 141.1	378.3 ± 130.1	−12.3 (−18.9 to −5.6)	−3.1%
Attenuation (HU)	TRO-CTA	374.5 ± 89.4	369.7 ± 84.0	−4.7 (−10.2 to 0.7)	−1.3%
Noise (HU)	CCTA	44.3 ± 9.0	22.8 ± 8.8	−21.5 (−23.2 to −19.9)	−48.6%
Noise (HU)	TRO-CTA	46.9 ± 8.0	25.2 ± 6.3	−21.7 (−24.3 to −19.2)	−46.3%
SNR	CCTA	9.5 ± 2.9	21.9 ± 6.4	12.4 (10.6 to 14.2)	131.3%
SNR	TRO-CTA	8.6 ± 2.0	19.6 ± 4.7	11.0 (9.7 to 12.4)	128.1%
CNR	CCTA	15.4 ± 5.1	41.6 ± 16.3	26.2 (20.3 to 32.1)	170.6%
CNR	TRO-CTA	14.3 ± 3.5	48.9 ± 16.0	34.5 (29.5 to 39.5)	240.6%

Values are mean ± SD. Standard CCTA *n* = 28; TRO-CTA *n* = 38. All within-protocol paired *p* values are <0.001 except TRO-CTA attenuation (*p* = 0.086). These subgroup tests are exploratory. Full interaction coefficients and adjusted *p* values are in Table S2. SD, standard deviation; AI, artificial intelligence; CCTA, coronary computed tomography angiography; CI, confidence interval; CNR, contrast-to-noise ratio; HIR, hybrid iterative reconstruction; HU, Hounsfield units; SNR, signal-to-noise ratio; TRO-CTA, triple-rule-out computed tomography angiography.

**Table 5:** Exploratory geometric percentage changes by subgroup.

Subgroup	n	Noise Change (95% CI)	SNR Change (95% CI)	CNR Change (95% CI)
Overall	66	−48.8% (−51.6 to −45.8)	130.1% (117.3 to 143.6)	201.7% (174.0 to 232.1)
Standard CCTA	28	−50.8% (−54.7 to −46.6)	134.6% (115.4 to 155.5)	166.6% (126.5 to 213.7)
TRO-CTA	38	−47.3% (−51.2 to −43.0)	126.8% (109.4 to 145.7)	230.4% (195.0 to 270.1)
HR < 70	26	−46.6% (−50.5 to −42.4)	126.4% (108.0 to 146.4)	220.6% (177.5 to 270.5)
HR ≥ 70	40	−50.2% (−54.0 to −46.0)	132.5% (114.8 to 151.7)	189.9% (154.3 to 230.6)
BMI < 25	47	−48.3% (−51.5 to −44.9)	124.8% (110.2 to 140.3)	174.8% (145.9 to 207.2)
BMI ≥ 25	19	−50.0% (−55.8 to −43.4)	143.8% (117.1 to 173.8)	279.8% (224.9 to 343.9)
CACS < 400	47	−48.8% (−52.3 to −45.1)	130.3% (115.2 to 146.5)	194.9% (164.0 to 229.3)
CACS ≥ 400	19	−48.7% (−53.5 to −43.4)	129.6% (104.3 to 158.0)	219.1% (159.5 to 292.5)

Geometric change = 100 × {exp[mean ln(AI/HIR)] – 1}; Cis are derived on the log scale. This differs from the arithmetic change in Table 2 and Table 4. Effects describe quantitative image-quality changes in measurable coronary lumina. AI, artificial intelligence; BMI, body mass index; CACS, coronary artery calcium score; CCTA, coronary computed tomography angiography; CI, confidence interval; CNR, contrast-to-noise ratio; HIR, hybrid iterative reconstruction; HR, heart rate; SNR, signal-to-noise ratio; TRO-CTA, triple-rule-out computed tomography angiography.

## Data Availability

The de-identified data that support the findings are available from the corresponding author upon reasonable request, subject to institutional and ethical restrictions.

## Supplementary Materials

The following supporting information can be downloaded at https://www.mdpi.com/article/10.3390/jcm15207940/s1, Table S1A–D (ROI comparisons), Table S2A–D (complete ROI-level models), Table S3A–C (patient-level log-ratio models), Table S4 (alternative noise-reference sensitivity), Table S5A–C (reader cross-tabulations and protocol-specific ratings), and Table S6 (acquisition and reconstruction specifications).

## Abbreviations

AI, artificial intelligence; BMI, body mass index; CABG, coronary artery bypass grafting; CAD, coronary artery disease; CACS, coronary artery calcium score; CCTA, coronary computed tomography angiography; CT, computed tomography; CI, confidence interval; CNR, contrast-to-noise ratio; CTDIvol, volume CT dose index; DLP, dose-length product; DLR, deep-learning reconstruction; ECG, electrocardiography; FOV, field of view; HIR, hybrid iterative reconstruction; HR, heart rate; HU, Hounsfield unit; IQR, interquartile range; LAD, left anterior descending artery; LCx, left circumflex artery; PACS, picture archiving and communication system; PCI, percutaneous coronary intervention; RCA, right coronary artery; ROI, region of interest; SD, standard deviation; SNR, signal-to-noise ratio; STROBE, Strengthening the Reporting of Observational Studies in Epidemiology; TRO-CTA, triple-rule-out computed tomography angiography.
