# Supplementary material for: Cardiac-Specific AI Reconstruction Versus Hybrid Iterative Reconstruction for Emergency Coronary CT Angiography: A Paired Single-Center Study Including Triple-Rule-Out CT
Source: J Clin Med. 2026 Oct 14;15(20):7940. doi: 10.3390/jcm15207940 (PMC13706594; doi:10.3390/jcm15207940)
Supplement: Supplementary file 1 [file jcm-15-07940-s001.zip › jcm-4578721-supplementary.pdf]

Supplementary Materials

Cardiac-Specific AI Reconstruction Versus Hybrid Iterative Reconstruction for Emergency Coronary CT Angiography: A Paired Single-Center Study Including Triple-Rule-Out CT

Manuscript JCM 4578721

These supplementary tables report complete paired estimates, model coefficients, sensitivity analyses, independent reader cross-tabulations, and technical specifications. The analysis includes 66 patients and 590 complete coronary region-of-interest (ROI) pairs. All confidence intervals are nominal 95% intervals unless specified.

Table S1A. Region-of-interest-specific paired comparisons of attenuation (Hounsfield units [HU]).

ROI	n	HIR	AI	Difference (95% CI)	Raw p	Holm p
LM	66	434.8 ± 134.5	425.9 ± 124.5	−8.9 (−20.8 to 2.9)	0.137	0.684
proximal RCA	66	436.9 ± 141.0	424.9 ± 133.3	−12.1 (−16.8 to −7.4)	<0.001	<0.001
proximal LAD	66	394.7 ± 126.5	388.7 ± 117.8	−6.0 (−14.4 to 2.5)	0.163	0.684
proximal LCx	66	396.7 ± 122.1	390.0 ± 112.8	−6.7 (−13.6 to 0.1)	0.054	0.324
mid RCA	64	396.9 ± 149.3	377.9 ± 131.4	−19.0 (−27.1 to −10.9)	<0.001	<0.001
mid LAD	66	364.3 ± 127.5	345.6 ± 113.7	−18.7 (−25.7 to −11.7)	<0.001	<0.001
distal RCA	65	401.0 ± 158.3	401.6 ± 142.9	0.6 (−7.0 to 8.2)	0.871	1.000
distal LAD	65	291.6 ± 111.4	287.7 ± 104.2	−3.8 (−10.4 to 2.7)	0.248	0.745
distal LCx	66	312.8 ± 124.1	314.6 ± 121.2	1.8 (−5.1 to 8.7)	0.599	1.000

Values are mean ± SD. Matched paired t comparisons. Holm adjustment is across nine locations separately for each outcome. Proximal/mid/distal identify coronary location. Different n values reflect complete pair availability. CIs are not adjusted for multiplicity. Abbreviations: AI, artificial intelligence; HIR, hybrid iterative reconstruction; ROI, region of interest; CI, confidence interval; SD, standard deviation; LM, left main; RCA, right coronary artery; LAD, left anterior descending artery; LCx, left circumflex artery; HU, Hounsfield units.

Primary patient-level estimates weight patients equally. ROI models and these location-specific tables are sensitivity analyses, not independent replications of the same patients.

Table S1B. Region-of-interest-specific paired comparisons of noise (Hounsfield units [HU]).

ROI	n	HIR	AI	Difference (95% CI)	Raw p	Holm p
LM	66	44.6 ± 10.2	15.5 ± 9.4	−29.2 (−31.5 to −26.9)	<0.001	<0.001
proximal RCA	66	44.0 ± 11.5	19.2 ± 9.5	−24.8 (−27.1 to −22.5)	<0.001	<0.001
proximal LAD	66	47.1 ± 14.3	26.1 ± 14.6	−21.1 (−24.2 to −17.9)	<0.001	<0.001
proximal LCx	66	48.6 ± 13.8	22.0 ± 12.6	−26.6 (−29.3 to −23.8)	<0.001	<0.001
mid RCA	64	44.4 ± 13.7	23.3 ± 11.6	−21.0 (−24.6 to −17.4)	<0.001	<0.001
mid LAD	66	43.7 ± 14.9	29.8 ± 14.5	−14.0 (−17.3 to −10.6)	<0.001	<0.001
distal RCA	65	51.3 ± 32.7	23.3 ± 10.8	−27.9 (−36.0 to −19.9)	<0.001	<0.001
distal LAD	65	43.2 ± 19.3	31.9 ± 20.1	−11.3 (−14.8 to −7.8)	<0.001	<0.001
distal LCx	66	44.9 ± 15.1	26.3 ± 16.5	−18.6 (−21.8 to −15.4)	<0.001	<0.001

Values are mean ± SD. Matched paired t comparisons. Holm adjustment is across nine locations separately for each outcome. Proximal/mid/distal identify coronary location. Different n values reflect complete pair availability. CIs are not adjusted for multiplicity. Abbreviations: AI, artificial intelligence; HIR, hybrid iterative reconstruction; ROI, region of interest; CI, confidence interval; SD, standard deviation; LM, left main; RCA, right coronary artery; LAD, left anterior descending artery; LCx, left circumflex artery; HU, Hounsfield units.

Table S1C. Region-of-interest-specific paired comparisons of signal-to-noise ratio (SNR).

ROI	n	HIR	AI	Difference (95% CI)	Raw p	Holm p
LM	66	10.0 ± 3.2	33.5 ± 15.2	23.5 (20.1 to 26.9)	<0.001	<0.001
proximal RCA	66	10.4 ± 4.0	26.5 ± 13.5	16.1 (13.4 to 18.9)	<0.001	<0.001
proximal LAD	66	9.0 ± 3.7	19.5 ± 10.8	10.5 (8.2 to 12.8)	<0.001	<0.001
proximal LCx	66	8.6 ± 3.1	22.6 ± 12.5	14.0 (11.2 to 16.8)	<0.001	<0.001
mid RCA	64	9.6 ± 4.3	20.3 ± 12.4	10.8 (8.1 to 13.4)	<0.001	<0.001
mid LAD	66	9.3 ± 4.8	15.3 ± 13.0	6.0 (3.5 to 8.5)	<0.001	<0.001
distal RCA	65	8.6 ± 3.6	20.1 ± 10.6	11.5 (9.2 to 13.7)	<0.001	<0.001
distal LAD	65	7.6 ± 4.2	11.7 ± 6.9	4.0 (2.6 to 5.5)	<0.001	<0.001
distal LCx	66	7.6 ± 3.8	15.7 ± 10.5	8.1 (5.8 to 10.4)	<0.001	<0.001

Values are mean ± SD. Matched paired t comparisons. Holm adjustment is across nine locations separately for each outcome. Proximal/mid/distal identify coronary location. Different n values reflect complete pair availability. CIs are not adjusted for multiplicity. Abbreviations: AI, artificial intelligence; HIR, hybrid iterative reconstruction; ROI, region of interest; CI, confidence interval; SD, standard deviation; LM, left main; RCA, right coronary artery; LAD, left anterior descending artery; LCx, left circumflex artery; SNR, signal-to-noise ratio.

Table S1D. Region-of-interest-specific paired comparisons of contrast-to-noise ratio (CNR).

ROI	n	HIR	AI	Difference (95% CI)	Raw p	Holm p
LM	66	16.4 ± 4.9	51.7 ± 20.5	35.3 (30.6 to 40.0)	<0.001	<0.001
proximal RCA	66	16.5 ± 5.1	50.9 ± 19.8	34.4 (29.9 to 38.9)	<0.001	<0.001
proximal LAD	66	15.2 ± 4.6	47.3 ± 17.8	32.2 (28.1 to 36.2)	<0.001	<0.001
proximal LCx	66	15.3 ± 4.6	47.3 ± 17.3	32.1 (28.1 to 36.0)	<0.001	<0.001
mid RCA	64	15.2 ± 4.9	46.1 ± 19.2	30.9 (26.6 to 35.3)	<0.001	<0.001
mid LAD	66	14.3 ± 4.8	42.6 ± 15.7	28.4 (24.7 to 32.0)	<0.001	<0.001
distal RCA	65	15.4 ± 5.4	47.9 ± 18.9	32.6 (28.4 to 36.7)	<0.001	<0.001
distal LAD	65	12.1 ± 4.1	36.9 ± 13.4	24.9 (21.7 to 28.0)	<0.001	<0.001
distal LCx	66	12.6 ± 4.4	39.7 ± 15.6	27.0 (23.6 to 30.5)	<0.001	<0.001

Values are mean ± SD. Matched paired t comparisons. Holm adjustment is across nine locations separately for each outcome. Proximal/mid/distal identify coronary location. Different n values reflect complete pair availability. CIs are not adjusted for multiplicity. Abbreviations: AI, artificial intelligence; HIR, hybrid iterative reconstruction; ROI, region of interest; CI, confidence interval; SD, standard deviation; LM, left main; RCA, right coronary artery; LAD, left anterior descending artery; LCx, left circumflex artery; CNR, contrast-to-noise ratio.

Table S2A. Complete region-of-interest-level model for attenuation (Hounsfield units [HU]).

Term	Estimate	SE	95% CI	Raw p	Holm p
Intercept	443.802	28.509	386.866 to 500.738	<0.001	—
AI	−12.438	3.155	−18.740 to −6.136	<0.001	—
TRO-CTA	−16.333	30.332	−76.911 to 44.246	0.592	—
AI × TRO-CTA	7.594	4.135	−0.664 to 15.853	0.071	0.213
proximal RCA	0.529	9.105	−17.654 to 18.712	0.954	—
proximal LAD	−38.689	8.949	−56.562 to −20.815	<0.001	—
proximal LCx	−37.036	9.689	−56.387 to −17.686	<0.001	—
mid RCA	−43.157	9.128	−61.385 to −24.928	<0.001	—
mid LAD	−75.374	13.053	−101.442 to −49.306	<0.001	—
distal RCA	−29.141	11.971	−53.049 to −5.233	0.018	—
distal LAD	−140.595	12.158	−164.876 to −116.314	<0.001	—
distal LCx	−116.702	13.304	−143.273 to −90.132	<0.001	—

Reference levels: HIR, standard CCTA, and left main ROI. N = 1180 measurements from 66 patients; 12 coefficients. Patient-clustered covariance with a finite-sample correction (CR1) uses 66/65 × 1179/1168 = 1.024947; t inference uses 65 degrees of freedom. The AI term is the AI–HIR effect for standard CCTA. AI × TRO-CTA estimates the additional effect in TRO-CTA. Holm p values apply only to the four protocol-interaction tests; other coefficients show nominal p values. CIs are nominal. Abbreviations: AI, artificial intelligence; HIR, hybrid iterative reconstruction; ROI, region of interest; SE, standard error; CI, confidence interval; CCTA, coronary computed tomography angiography; TRO-CTA, triple-rule-out computed tomography angiography; RCA, right coronary artery; LAD, left anterior descending artery; LCx, left circumflex artery; HU, Hounsfield units.

Table S2B. Complete region-of-interest-level model for noise (Hounsfield units [HU]).

Term	Estimate	SE	95% CI	Raw p	Holm p
Intercept	39.375	1.529	36.321 to 42.428	<0.001	—
AI	−21.510	0.802	−23.111 to −19.908	<0.001	—
TRO-CTA	2.593	2.139	−1.679 to 6.864	0.230	—
AI × TRO-CTA	−0.188	1.501	−3.186 to 2.810	0.901	0.901
proximal RCA	1.509	1.116	−0.719 to 3.737	0.181	—
proximal LAD	6.559	1.663	3.238 to 9.879	<0.001	—
proximal LCx	5.231	1.346	2.542 to 7.920	<0.001	—
mid RCA	3.824	1.582	0.665 to 6.983	0.018	—
mid LAD	6.692	1.931	2.835 to 10.549	<0.001	—
distal RCA	7.273	2.444	2.392 to 12.155	0.004	—
distal LAD	7.490	2.425	2.648 to 12.332	0.003	—
distal LCx	5.543	2.027	1.496 to 9.591	0.008	—

Reference levels: HIR, standard CCTA, and left main ROI. N = 1180 measurements from 66 patients; 12 coefficients. Patient-clustered covariance with a finite-sample correction (CR1) uses 66/65 × 1179/1168 = 1.024947; t inference uses 65 degrees of freedom. The AI term is the AI–HIR effect for standard CCTA. AI × TRO-CTA estimates the additional effect in TRO-CTA. Holm p values apply only to the four protocol-interaction tests; other coefficients show nominal p values. CIs are nominal. Abbreviations: AI, artificial intelligence; HIR, hybrid iterative reconstruction; ROI, region of interest; SE, standard error; CI, confidence interval; CCTA, coronary computed tomography angiography; TRO-CTA, triple-rule-out computed tomography angiography; RCA, right coronary artery; LAD, left anterior descending artery; LCx, left circumflex artery; HU, Hounsfield units.

Table S2C. Complete region-of-interest-level model for signal-to-noise ratio (SNR).

Term	Estimate	SE	95% CI	Raw p	Holm p
Intercept	16.451	1.076	14.301 to 18.600	<0.001	—
AI	12.423	0.867	10.692 to 14.154	<0.001	—
TRO-CTA	−0.860	0.633	−2.124 to 0.405	0.179	—
AI × TRO-CTA	−1.396	1.098	−3.588 to 0.796	0.208	0.416
proximal RCA	−3.296	1.223	−5.738 to −0.854	0.009	—
proximal LAD	−7.545	1.226	−9.994 to −5.095	<0.001	—
proximal LCx	−6.179	1.066	−8.308 to −4.049	<0.001	—
mid RCA	−6.848	1.276	−9.396 to −4.299	<0.001	—
mid LAD	−9.468	1.352	−12.168 to −6.767	<0.001	—
distal RCA	−7.406	1.181	−9.766 to −5.047	<0.001	—
distal LAD	−12.099	1.177	−14.451 to −9.748	<0.001	—
distal LCx	−10.126	1.280	−12.683 to −7.570	<0.001	—

Reference levels: HIR, standard CCTA, and left main ROI. N = 1180 measurements from 66 patients; 12 coefficients. Patient-clustered covariance with a finite-sample correction (CR1) uses 66/65 × 1179/1168 = 1.024947; t inference uses 65 degrees of freedom. The AI term is the AI–HIR effect for standard CCTA. AI × TRO-CTA estimates the additional effect in TRO-CTA. Holm p values apply only to the four protocol-interaction tests; other coefficients show nominal p values. CIs are nominal. Abbreviations: AI, artificial intelligence; HIR, hybrid iterative reconstruction; ROI, region of interest; SE, standard error; CI, confidence interval; CCTA, coronary computed tomography angiography; TRO-CTA, triple-rule-out computed tomography angiography; RCA, right coronary artery; LAD, left anterior descending artery; LCx, left circumflex artery; SNR, signal-to-noise ratio.

Table S2D. Complete region-of-interest-level model for contrast-to-noise ratio (CNR).

Term	Estimate	SE	95% CI	Raw p	Holm p
Intercept	19.247	1.118	17.013 to 21.480	<0.001	—
AI	26.127	2.837	20.461 to 31.792	<0.001	—
TRO-CTA	−1.024	1.119	−3.259 to 1.212	0.364	—
AI × TRO-CTA	8.244	3.732	0.790 to 15.697	0.031	0.123
proximal RCA	−0.369	0.584	−1.536 to 0.797	0.529	—
proximal LAD	−2.842	0.625	−4.090 to −1.595	<0.001	—
proximal LCx	−2.793	0.698	−4.187 to −1.399	<0.001	—
mid RCA	−3.378	0.653	−4.683 to −2.074	<0.001	—
mid LAD	−5.631	0.858	−7.345 to −3.917	<0.001	—
distal RCA	−2.423	0.786	−3.992 to −0.854	0.003	—
distal LAD	−9.611	0.904	−11.415 to −7.806	<0.001	—
distal LCx	−7.932	1.020	−9.968 to −5.895	<0.001	—

Reference levels: HIR, standard CCTA, and left main ROI. N = 1180 measurements from 66 patients; 12 coefficients. Patient-clustered covariance with a finite-sample correction (CR1) uses 66/65 × 1179/1168 = 1.024947; t inference uses 65 degrees of freedom. The AI term is the AI–HIR effect for standard CCTA. AI × TRO-CTA estimates the additional effect in TRO-CTA. Holm p values apply only to the four protocol-interaction tests; other coefficients show nominal p values. CIs are nominal. Abbreviations: AI, artificial intelligence; HIR, hybrid iterative reconstruction; ROI, region of interest; SE, standard error; CI, confidence interval; CCTA, coronary computed tomography angiography; TRO-CTA, triple-rule-out computed tomography angiography; RCA, right coronary artery; LAD, left anterior descending artery; LCx, left circumflex artery; CNR, contrast-to-noise ratio.

Table S3A. Patient-level effect-modification model for noise.

Term	Log-scale coefficient	95% CI	Ratio of ratios (95% CI)	Raw p	Holm p
Intercept	0.120	−0.667 to 0.906	1.127 (0.513 to 2.475)	0.762	—
TRO-CTA	0.141	0.018 to 0.263	1.151 (1.019 to 1.301)	0.025	0.349
BMI per 5 kg/m²	−0.095	−0.218 to 0.029	0.910 (0.804 to 1.029)	0.131	1.000
Heart rate per 10 bpm	−0.036	−0.083 to 0.011	0.965 (0.921 to 1.011)	0.127	1.000
CTDIvol per 5 mGy	−0.048	−0.116 to 0.020	0.953 (0.890 to 1.020)	0.160	1.000
ln(1 + calcium score)	0.010	−0.009 to 0.029	1.010 (0.991 to 1.030)	0.285	1.000

Outcome: ln(patient AI/HIR ratio); n = 66, type 3 heteroscedasticity-consistent covariance (HC3), t inference with 60 degrees of freedom. Standard CCTA is the protocol reference. The multiplicative ratio is exp(coefficient); for modifiers it describes a change in the reconstruction ratio, not the AI/HIR ratio itself. Calcium enters as ln(1 + score), retaining 30 zero values. Holm adjustment includes five modifier tests in each of three models (15 tests). The intercept is shown for completeness. CIs are nominal and may exclude the null despite a nonsignificant adjusted p value. Abbreviations: AI, artificial intelligence; HIR, hybrid iterative reconstruction; CI, confidence interval; CCTA, coronary computed tomography angiography; TRO-CTA, triple-rule-out computed tomography angiography; BMI, body mass index; CTDIvol, volume computed tomography dose index; bpm, beats per minute; ln, natural logarithm; exp, exponential function.

Table S3B. Patient-level effect-modification model for signal-to-noise ratio (SNR).

Term	Log-scale coefficient	95% CI	Ratio of ratios (95% CI)	Raw p	Holm p
Intercept	0.004	−0.685 to 0.694	1.004 (0.504 to 2.001)	0.990	—
TRO-CTA	−0.110	−0.236 to 0.017	0.896 (0.790 to 1.017)	0.088	0.965
BMI per 5 kg/m²	0.109	−0.003 to 0.221	1.115 (0.997 to 1.247)	0.056	0.675
Heart rate per 10 bpm	0.021	−0.015 to 0.057	1.021 (0.986 to 1.059)	0.241	1.000
CTDIvol per 5 mGy	0.062	0.008 to 0.116	1.064 (1.008 to 1.123)	0.026	0.349
ln(1 + calcium score)	−0.005	−0.025 to 0.014	0.995 (0.975 to 1.015)	0.583	1.000

Outcome: ln(patient AI/HIR ratio); n = 66, type 3 heteroscedasticity-consistent covariance (HC3), t inference with 60 degrees of freedom. Standard CCTA is the protocol reference. The multiplicative ratio is exp(coefficient); for modifiers it describes a change in the reconstruction ratio, not the AI/HIR ratio itself. Calcium enters as ln(1 + score), retaining 30 zero values. Holm adjustment includes five modifier tests in each of three models (15 tests). The intercept is shown for completeness. CIs are nominal and may exclude the null despite a nonsignificant adjusted p value. Abbreviations: AI, artificial intelligence; HIR, hybrid iterative reconstruction; CI, confidence interval; CCTA, coronary computed tomography angiography; TRO-CTA, triple-rule-out computed tomography angiography; BMI, body mass index; CTDIvol, volume computed tomography dose index; bpm, beats per minute; ln, natural logarithm; exp, exponential function; SNR, signal-to-noise ratio.

Table S3C. Patient-level effect-modification model for contrast-to-noise ratio (CNR).

Term	Log-scale coefficient	95% CI	Ratio of ratios (95% CI)	Raw p	Holm p
Intercept	−0.032	−0.919 to 0.855	0.968 (0.399 to 2.350)	0.942	—
TRO-CTA	0.123	−0.082 to 0.328	1.131 (0.921 to 1.388)	0.236	1.000
BMI per 5 kg/m²	0.174	0.041 to 0.308	1.191 (1.042 to 1.361)	0.011	0.169
Heart rate per 10 bpm	0.006	−0.060 to 0.072	1.006 (0.942 to 1.075)	0.849	1.000
CTDIvol per 5 mGy	0.036	−0.066 to 0.137	1.036 (0.936 to 1.147)	0.485	1.000
ln(1 + calcium score)	0.018	−0.013 to 0.049	1.018 (0.987 to 1.050)	0.248	1.000

Outcome: ln(patient AI/HIR ratio); n = 66, type 3 heteroscedasticity-consistent covariance (HC3), t inference with 60 degrees of freedom. Standard CCTA is the protocol reference. The multiplicative ratio is exp(coefficient); for modifiers it describes a change in the reconstruction ratio, not the AI/HIR ratio itself. Calcium enters as ln(1 + score), retaining 30 zero values. Holm adjustment includes five modifier tests in each of three models (15 tests). The intercept is shown for completeness. CIs are nominal and may exclude the null despite a nonsignificant adjusted p value. Abbreviations: AI, artificial intelligence; HIR, hybrid iterative reconstruction; CI, confidence interval; CCTA, coronary computed tomography angiography; TRO-CTA, triple-rule-out computed tomography angiography; BMI, body mass index; CTDIvol, volume computed tomography dose index; bpm, beats per minute; ln, natural logarithm; exp, exponential function; CNR, contrast-to-noise ratio.

Table S4. Sensitivity to the noise-reference denominator.

Noise reference	HIR	AI	Paired difference (95% CI)	Arithmetic change	Raw p
Pericardial-fat SD (primary CNR)	14.8 ± 4.2	45.8 ± 16.4	31.0 (27.2 to 34.8)	209.7%	<0.001
Aortic blood-pool SD	12.5 ± 3.9	33.1 ± 11.9	20.6 (18.4 to 22.7)	164.2%	<0.001
Left-ventricular SD	11.4 ± 3.6	32.1 ± 11.9	20.7 (18.1 to 23.4)	182.2%	<0.001

Each index retains the same patient-level coronary-minus-pericardial-fat attenuation numerator; only the within-tissue noise denominator changes. Paired t comparisons, n = 66. Alternative indices are exploratory sensitivity measures and should not be treated as validated lesion-detectability metrics. The direction persists, but the magnitude depends on the denominator. Values are mean ± SD. Abbreviations: AI, artificial intelligence; HIR, hybrid iterative reconstruction; CI, confidence interval; SD, standard deviation; CNR, contrast-to-noise ratio.

Supportive checks for the three co-primary patient-level outcomes.

Outcome	Cohen dz	Paired t p	Holm p	Wilcoxon p
Noise (HU)	−3.33	<0.001	<0.001	<0.001
SNR	2.66	<0.001	<0.001	<0.001
CNR	1.99	<0.001	<0.001	<0.001

Cohen dz = mean paired difference / SD of paired differences. Holm correction applies to the three co-primary paired-t tests; Wilcoxon tests are supportive sensitivity analyses. Abbreviations: SD, standard deviation; HU, Hounsfield units; SNR, signal-to-noise ratio; CNR, contrast-to-noise ratio.

Table S5A. Independent reader cross-tabulation for overall image quality.

Reader 1 / Reader 2	HIR superior	No preference	AI superior	Total
HIR superior	0	0	0	0
No preference	0	0	1	1
AI superior	0	3	62	65
Reader 2 total	0	3	63	66

Exact agreement 93.9% (Wilson 95% CI, 85.4 to 97.6%); Cohen κ −0.023. Bootstrap 95% CI, −0.055 to 0.000. Abbreviations: AI, artificial intelligence; HIR, hybrid iterative reconstruction; CI, confidence interval; κ, Cohen kappa.

Table S5B. Independent reader cross-tabulation for perceived vessel-edge sharpness.

Reader 1 / Reader 2	HIR superior	No preference	AI superior	Total
HIR superior	0	0	0	0
No preference	0	1	1	2
AI superior	0	2	62	64
Reader 2 total	0	3	63	66

Exact agreement 95.5% (Wilson 95% CI, 87.5 to 98.4%); Cohen κ 0.377. Bootstrap 95% CI, −0.038 to 1.000. Abbreviations: AI, artificial intelligence; HIR, hybrid iterative reconstruction; CI, confidence interval; κ, Cohen kappa.

Each reader independently rated the same 66 paired examinations during revision. There was no consensus conversion. κ intervals use 20,000 patient-level bootstrap draws per criterion (seed 20261006), excluding undefined estimates: 19,671 quality draws and 19,693 sharpness draws were valid. For quality, expected agreement was 94.1%, slightly above observed agreement (93.9%). Ratings were concentrated in the AI-superior category, and no joint no-preference ratings occurred for quality. κ and its bootstrap intervals are sample-dependent and do not establish reproducibility across a broader range of image quality.

Table S5C. Reader-specific results stratified by protocol.

Protocol	Criterion	Reader	n	AI superior	No preference	HIR superior
CCTA	Quality	1	28	27 (96.4%)	1 (3.6%)	0 (0.0%)
CCTA	Quality	2	28	26 (92.9%)	2 (7.1%)	0 (0.0%)
CCTA	Sharpness	1	28	26 (92.9%)	2 (7.1%)	0 (0.0%)
CCTA	Sharpness	2	28	27 (96.4%)	1 (3.6%)	0 (0.0%)
TRO-CTA	Quality	1	38	38 (100.0%)	0 (0.0%)	0 (0.0%)
TRO-CTA	Quality	2	38	37 (97.4%)	1 (2.6%)	0 (0.0%)
TRO-CTA	Sharpness	1	38	38 (100.0%)	0 (0.0%)	0 (0.0%)
TRO-CTA	Sharpness	2	38	36 (94.7%)	2 (5.3%)	0 (0.0%)

Protocol-specific descriptions are exploratory. All two-sided sign tests excluding ties were $p < 0.001$. These tests summarize comparative preference, do not assess reader agreement, and do not establish absolute diagnostic adequacy. Abbreviations: AI, artificial intelligence; HIR, hybrid iterative reconstruction; CCTA, coronary computed tomography angiography; TRO-CTA, triple-rule-out computed tomography angiography.

Table S6. Acquisition and reconstruction specifications.

Item	Specification
Scanner / software	uCT 960+ / uExceed R001
Coronary acquisition	Prospective ECG-triggered axial; 320 × 0.5-mm detector rows
Tube settings / rotation	100 kVp; fixed 180 mAs; 0.25 s
Acquisition window	20%–80% of the R–R interval
Reconstruction phase	Identical within pairs; mid-diastole 47, end-systole 19
Motion correction	CardioCapture setting 5, identical within pairs
AI reconstruction	CardioBoost noise-reduction level 2
HIR reconstruction	KARL3D strength 6; C_SOFT_DA kernel
AI kernel	No separately selectable user kernel
Setting rationale	Manufacturer-recommended settings used in the local workflow
Matrix / thickness / increment	512 × 512 / 0.5 mm / 0.25 mm
Numerical reconstruction FOV	Typically 140 × 140 mm; adjusted to patient heart size
Postprocessing and ROI placement	Offline Vitrea workstation for postprocessing; ZeTTA PACS for image review and matched ROI copy/paste
Coronary ROI area (mean ± SD)	7.0 ± 3.1 mm^2
Postprocessing/PACS version	Vitrea 7.12 (Vital Images, Minnetonka, MN, USA); ZeTTA PACS 2.0.3.10 (TaeYoung Soft, Gwacheon, Republic of Korea)
Heart-rate medication / nitroglycerin	No pharmacologic heart-rate control; nitroglycerin in all 66
Standard CCTA injection	80 mL contrast + 30 mL saline, 5 mL/s
TRO test injection	15 mL contrast + 25 mL saline, 5 mL/s
TRO diagnostic injection	60 mL contrast + 30 mL 60:40 contrast/saline + 40 mL saline, 5 mL/s
Total protocol contrast volume	CCTA 80 mL; TRO 93 mL including test bolus
Contrast agent	Iohexol 350 mg iodine/mL
Radiation reporting scope	Coronary acquisition only; not total TRO examination

Specifications describe the investigated configuration. Identical paired phases and motion settings were confirmed; no strength-optimization, dose-reduction, or cross-vendor comparison was performed. Abbreviations: AI, artificial intelligence; HIR, hybrid iterative reconstruction; ECG, electrocardiogram; R–R, interval between consecutive R waves; CCTA, coronary computed tomography angiography; TRO, triple rule out; ROI, region of interest; SD, standard deviation; FOV, field of view; MPR, multiplanar reformation; PACS, picture archiving and communication system; kVp, peak kilovoltage; mAs, milliampere-seconds.
